# Phosphorylation of Single Stranded RNA Virus Proteins and Potential for Novel Therapeutic Strategies

**DOI:** 10.3390/v7102872

**Published:** 2015-10-12

**Authors:** Forrest Keck, Pouya Ataey, Moushimi Amaya, Charles Bailey, Aarthi Narayanan

**Affiliations:** National Center for Biodefense and Infectious Disease, School of Systems Biology, George Mason University, 10650 Pyramid Place, Manassas, VA 20110, USA; fkeck@masonlive.gmu.edu (F.K.); pataey@gmu.edu (P.A.); mamaya3@gmu.edu (M.A.); cbailey2@gmu.edu (C.B.)

**Keywords:** post translational modification, viral protein, phosphorylation, therapeutics, kinase, antivirals, J0101

## Abstract

Post translational modification of proteins is a critical requirement that regulates function. Among the diverse kinds of protein post translational modifications, phosphorylation plays essential roles in protein folding, protein:protein interactions, signal transduction, intracellular localization, transcription regulation, cell cycle progression, survival and apoptosis. Protein phosphorylation is also essential for many intracellular pathogens to establish a productive infection cycle. Preservation of protein phosphorylation moieties in pathogens in a manner that mirrors the host components underscores the co-evolutionary trajectory of pathogens and hosts, and sheds light on how successful pathogens have usurped, either in part or as a whole, the host enzymatic machinery. Phosphorylation of viral proteins for many acute RNA viruses including Flaviviruses and Alphaviruses has been demonstrated to be critical for protein functionality. This review focuses on phosphorylation modifications that have been documented to occur on viral proteins with emphasis on acutely infectious, single stranded RNA viruses. The review additionally explores the possibility of repurposing Food and Drug Administration (FDA) approved inhibitors as antivirals for the treatment of acute RNA viral infections.

## 1. Introduction

Post translational modifications are defined as alterations made to a protein at multiple stages after translation and generation of the nascent polypeptide chain. Such modifications that occur in prokaryotic and eukaryotic cells can take the form of appending of unique chemical moieties to specified side chains on the nascent polypeptide. The transfer of the chemical moiety occurs as an enzyme catalyzed event involving an electrophilic substrate transfer of the target protein. Post-translational modification may also involve protein cleavage/trimming from a premature to mature form mediated by proteases [1]. Usually, post-translational modifications are transient in nature with a constant flux of the target protein between the functionally active and functionally inactive states. Such a flux is the central component of functional regulation of proteins, which will impact protein stability, interaction partners and subcellular localization in a manner that transcends the properties of the nascent translated product.

Post translational modifications of pathogen-derived proteins can include phosphorylation, methylation, acetylation, glycosylation, ubiquitylation, Small Ubiquitin-like Modifier (SUMO)ylation and nitrosylation. These modifications have been subject to intense research because of the unique insights they can offer into host-pathogen interactions that can extend to the development of novel diagnostics and therapeutics. Many viral proteins have been demonstrated to be post translationally modified during various stages of the infectious process and involve enzymatic components that are derived from both the pathogen and the host [2]. In fact, one can envision a co-evolutionary strategy whereby viruses “learnt” to utilize the host enzymatic machinery that has already been activated as a part of the innate immune response. This learning process that spans multiple generations in the evolutionary scale continues to modify the viral landscape, more extensively so with pathogen-directed therapeutics driving some of the modifications in the quest for evolutionary fitness. In this review, we have focused on phosphorylation events that occur on viral proteins of single stranded RNA viruses as a part of acute viral infections. The review also includes insights into how such host-directed events have been exploited to lend support to rational design of small molecule therapeutics.

## 2. Protein Phosphorylation

**Figure 1:**
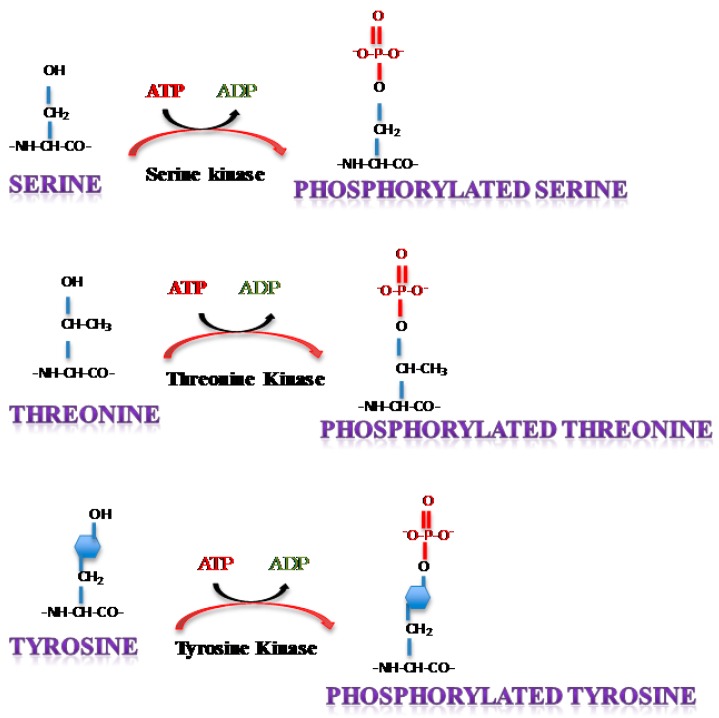
Post translational addition of a phosphorylation moiety on Serine, Threonine or Tyrosine residues. ATP: Adenosine triphosphate, ADP: Adenosine diphosphate.

Phosphorylation is the most common form of transient protein modification that has been shown to be imperative to biological activity by influencing different cellular processes including cell growth, differentiation and apoptosis. In yeast, flies and humans, an estimated 30% of the total proteome is considered to be phosphorylated [3,4]. This reversible event involves the addition of a phosphate group to serine (Ser), threonine (Thr) or tyrosine (Tyr) residues on proteins in a manner that requires adenosine triphosphate (ATP) (Figure 1).

Protein kinases constitute the enzymatic effectors that allow the transfer of a terminal phosphate group provided mostly but not exclusively from ATP to the nucleophile (OH) group of Serine, Threonine or Tyrosine. These mediator protein kinases are estimated to comprise 2% of the fly, yeast or human genomes (the kinome) and represent one of the largest functional classes of proteins [5]. Protein phosphorylation is considered to introduce a change in the charge of the acceptor protein which results in a conformational change and alters the activation status of the protein.

Phosphorylation events can contribute to signal transduction where a single protein is modified in response to a stimulus and results in an outcome. Eukaryotes demonstrate complex signal transduction cascades where the stimulus from the source is passed through an array of effector molecules that can either independently or synergistically influence diverse functions. Such transduction of the signal through multiple effectors or cascades helps to amplify the signal and diversify the host response. One paradigm example of such information transfer mediated by phosphorylation of multiple target proteins sequentially is the nuclear factor kappa-light-chain-enhancer of activated B cells (NFκB) cascade [6]. Multiple excellent reviews on NFκB cascade activation exist in the literature that bear testament to the finely tuned information transfer that mediates host transcriptional regulation. Signal transduction through Mitogen Activated Protein Kinase (MAPK) cascades constitutes another prominent example where multiple kinases are successively phosphorylated that ultimately leads to transcriptional activation involving a phosphorylated transcriptional regulatory protein [7].

Many post translationally phosphorylated proteins are modified on multiple sites which contributes to differential regulation of protein function. For example, the Heat Shock Factor (HSF) protein can be phosphorylated on a total of five possible sites. Phosphorylation of Ser230, Ser303, Ser307 and Ser363 is usually associated with a functionally inactive state when the protein exists as a monomer and transcriptionally repressed. Enhanced phosphorylation of Ser230 following a stress stimulus leads to HSF trimerization, DNA (promoter) binding and enhances transcriptional activity [8]. Another transcriptional factor that has been documented to be differentially regulated based on phosphorylation events is p53, which was recognized as a phosphoprotein many decades ago. To date, p53 is known to be phosphorylated on 16 sites with the vast majority being induced as a result of a stress stimulus, with a limited number of dephosphorylation events associated with protein activation [9]. The phosphorylation status of p53 induces protein stabilization, protein translocation to the nucleus, DNA binding and transcriptional activation. The effector molecule in the NFκB cascade, p65, is another hyperphosphorylated protein that is a key regulator of host transcription. p65 is phosphorylated on multiple residues (Ser276, Ser311, Ser468, Ser505, Ser529, Ser536) via multiple host kinases (PKAc, MSK1, GSK3β, Chk1, CK2, RSK1, IKKε, IKKα, IKKβ) and these events have different effects on nuclear translocation, promoter DNA binding and interaction with transcriptional activators [10,11,12,13]. There are numerous such examples in the literature of how eukaryotic proteins are phosphorylated by the host enzymatic machinery and are essential for maintaining cell viability and function. The focus of this review is phosphorylation of viral proteins, mediated by activated host kinases.

Viruses, being obligate parasites, have evolved to utilize the host kinase machinery to bring about phosphorylation modifications of viral proteins that impact establishment of a productive infection and control of host innate immunity. Interestingly, whole genome sequencing has revealed strong homology between viral encoded kinases and eukaryotic protein kinases, which attests to the ability of viral components to effectively sidetrack the host kinase machinery to mediate their replication strategy. Studies using mutants of the vaccinia virus identified viruses that were arrested during the DNA replication stage. Such mutants were found to have mutations located in the *B1* gene, sequencing of which revealed that the *B1* gene shows homology with human Ser/Thr kinases. Continued investigation confirmed that *B1* encoded catalytically active viral Ser/Thr kinases that led to the discovery of a novel class of Ser/Thr kinases shared by nearly all poxviruses and expressed early in infection [14,15,16]. In the following sections of this review, we will focus on phosphorylation of viral proteins documented for acutely infectious RNA viruses that cause disease. In addition, the review will provide examples of how such phosphorylation modifications can be utilized as targets for therapeutic intervention to control viral multiplication and increase host survival. We wish to emphasize at this juncture that all the viruses that are under discussion in this review have been recorded to induce host protein phosphorylation in infected cells. There are numerous evidences in published literature of host kinase cascades that are differentially phosphorylated in virus infected cells in a manner that will contribute to viral multiplication. Such events are not the subject of discussion and the focus will be exclusively on viral protein phosphorylation in infected host cells.

## 3. Phosphorylation of Single Stranded RNA Virus Proteins

Single stranded RNA (ssRNA) viruses are the most abundant of all virus genera and consist of both positive-strand RNA and negative-strand RNA viruses. Instances of virus protein phosphorylation has been shown, as mediated by diverse host kinases for both types of ssRNA viruses [17].

The positive sense ssRNA viruses that will be discussed as part of this review will include the following families: Flavivirus, Alphavirus, and Rubivirus. Positive sense ssRNA viruses have their genomic RNA translated to produce viral proteins, which includes both structural and non-structural proteins. The replication of the virus involves recruitment of both viral and host machinery including viral RNA-dependent RNA polymerase (RdRp) as one of the key components. RdRp uses the incoming positive-strand RNA as a template to create the complementary negative strand RNA, which is used for direct synthesis of progeny RNA molecules [18]. In these intricate steps, multiple viral proteins are post translationally modified that occupy unique niches in the viral replication cycle as will be discussed.

Negative sense ssRNA viruses that will be discussed in this article include the following: Filovirus, and Orthomyxovirus. In the case of negative sense ssRNA viruses, the genome must be transcribed by a single virally encoded RdRp into messenger RNA (mRNA), which in turn encodes individual viral proteins. The mRNA transcripts are initiated, elongated, terminated, capped, methylated, and polyadenylated all by RdRp which also produces a full length positive strand RNA for further copies of negative strand progeny genomes to be made.

### 3.1. Positive Sense ssRNA Viruses

#### 3.1.1. Flaviviruses

This review will cover four genera of the *Flaviviridae* family including three mosquito-borne viruses (Dengue, West Nile, and Yellow Fever) and one tick-borne virus (Tick-borne encephalitis). While an effective vaccine exists for Yellow Fever Virus (YFV), no vaccines or therapeutics exist for Dengue Virus (DENV) or West Nile Virus (WNV). The efficacies of cell culture derived Tick-borne encephalitis virus (TBEV) vaccines have been well documented and have been used for human vaccinations.

The flavivirus genome encodes three structural proteins (Capsid, Envelope, M protein) and seven nonstructural proteins (NS1–5). Non-structural protein 5 (NS5), the largest of the flaviviral nonstructural proteins, has been indicated to be phosphorylated at conserved Ser/Thr residues in the case of all above mentioned viruses [19]. NS5 includes a C-terminal domain consisting of 600 amino acids with RdRp activity and an N-terminal domain with 300 amino acids representing the methyltransferase domain involved in methylation of cap structures on the 5′ end of viral RNA. Both these activities are vital for the viral life cycle and replication of viral RNA. NS5 of flaviviruses has been extensively studied for its role in counteracting the host interferon response by modulating the phosphorylation of host signaling molecules, including Signal Transducer and Activator of Transcription (STAT) proteins [20]. It has been shown that phosphorylation of NS5 plays a role in ensuring that Janus kinase (JAK)/STAT signaling is effectively suppressed [21]. Since controlling the host responses mediated by STAT requires a phosphorylated NS5, one can envision a treatment rationale that can restore the natural innate immune responses of the host that might otherwise be controlled by the virus.

Phospho-acceptor motifs are conserved among DENV, WNV, and YFV NS5 proteins which are phosphorylated by cyclic guanosine monophosphate (cGMP)-dependent Protein Kinase G (PKG) *in vitro*, specifically on Thr449. *In vitro* kinase reactions of NS5 with PKG were analyzed by mass spectrometry to determine that the same threonine was phosphorylated both in cells and *in vitro*, which showed that the conserved region was accessible in both instances and phosphorylation can occur through any member of the PKG family. To determine the importance of Thr449, a polymorphism was introduced with the substitution of a positively charged histidine or negatively charged glutamic acid, both of which rendered the protein nonfunctional, while a serine substitution allowed nearly identical efficiency of replication in wild type-YFV and -DENV NS5 [22].

Forwood *et al.*, had demonstrated a novel nuclear localization signal (NLS) the linker region of DENV NS5 protein [23]. The linker domain housed a functional casein kinase (CK) 2 phosphorylation site (Thr395). The authors proposed that the site may inhibit nuclear targeting of NS5 by enabling cytoplasmic retention and hence may be involved in regulation of NS5 nuclear localization during the DENV infectious cycle [23].

DENV NS5 has been demonstrated to interact with NS3 in the cytoplasm of infected cells. This interaction between the two DENV NS proteins was dependent on NS5 phosphorylation [24]. NS3 is an interesting case because there is implication that flaviviral NS3 may be phosphorylated, particularly in the context of its polypeptide processing enzymatic function; however, no evidence exists thus far that directly demonstrate phosphorylation status of NS3. NS3 may afford an interesting and potent therapeutic target if phosphorylated residues and their mediator kinases are identified.

WNV NS5, in agreement with other flaviviral NS5 protein studies, has been indicated to be phosphorylated on serine and threonine residues, but not tyrosine [25]. This phosphorylation status was reported in WNV Kunjin strain infected vero cells, where it was noted that WNV NS5 does not translocate to the nucleus. Instead, this closely mirrors the hepatitis C virus RdRp NS5B protein, suggesting an important functional role of viral polymerase phosphorylation [25]. Further characterization of the cytoplasmic WNV NS5 phosphorylation is required to elucidate the specific role of the reported phosphorylation events.

Ser56 (S56) of YFV NS5 was shown to be phosphorylated by a combination of mass spectrometry and site-directed mutagenesis methods [26]. S56 is believed to interact with *S*-adenosyl homocysteine in the YFV NS5 methyltransferase domain by catalyzing the transfer of a methyl group from *S*-adenosyl-L-methionine (SAM) to the viral RNA. This enables the virus to evade RNA cap-based recognition and escape the innate immune response. The phosphorylation of NS5 on S56 is therefore an important requirement for evading cap-dependent innate immune events in the host cell. Sequence alignment and analysis of more than 700 flaviviral NS5 proteins show a high level of conservation of S56 and the surrounding residues, which typically create a kinase recognition sequence. With both mutational studies and additional mass spectrometry data, S56 was found to be completely conserved in the genus Flavivirus and essential for YFV RNA 2′-O methylation and replication [26]. Bhattacharya *et al.*, have also demonstrated that small molecule mediated inhibition of CK1 suppressed YFV multiplication, potentially by interfering with S56 phosphorylation mediated by the alpha isoform of CK1 (CK1α) [26]. The same kinase was also shown to be able to phosphorylate S56 of Hepatitis C virus (HCV) NS5A protein.

HCV NS5A regulates the replication of viral RNA and viral particle assembly in a Ser/Thr phosphorylation-dependent manner. Yamauchi *et al.*, demonstrated that HCV particle assembly requires phosphorylation of NS5A by the c-Abl tyrosine kinase, and knockdown of c-Abl reduces output of infectious HCV particles without impacting rates of RNA translation or replication. c-Abl was shown to phosphorylate residue Tyr(330) of NS5A in HCV-infected cells [27].

TBEV NS5 was demonstrated to be phosphorylated in infected cell extracts in the presence of [γ32P] ATP, as shown by a combination of sodium dodecyl sulfate polyacrylamide gel electrophoresis (SDS-PAGE) and thin layer chromatography. Phospho-amino acid analysis indicated that NS5 contains multiple phospho-serine sites, but no phospho-threonine, or phospho-tyrosine [28]. This activity was observed in the perinuclear endoplasmic reticulum (pER) membranes, where NS5 phosphorylation is thought to play a role in its interaction with NS3 during early infection, and later stage localization to the cytoplasm. Morozova *et al.*, suggested that phosphorylation could regulate TBEV genome replication and switching from minus-strand RNA synthesis to plus-strand synthesis [28].

#### 3.1.2. Alphaviruses

Alphaviruses are also arthropod-borne viruses (arboviruses) and are commonly transmitted by mosquito vectors. Alphaviruses are represented by new world alphaviruses and old world alphaviruses. The new world alphaviruses are encephalitic viruses and include Venezuelan Equine Encephalitis Virus (VEEV), Eastern Equine Encephalitis Virus (EEEV) and Western Equine Encephalitis Virus (WEEV). The old world alphaviruses cause an arthritic disease and include Semliki Forrest Virus (SFV), Sindbis Virus (SINV) and Chikungunya Virus (CHIKV). Both groups of alphaviruses can cause severe diseases in humans during natural epizootic outbreaks. While mosquito bites are the most common form of transmission, new world alphaviruses like VEEV are highly stable and infectious when transmitted through the aerosol route. Infections are typically acute and can produce high-titer viremia levels [29]. There are currently no FDA-approved vaccines or therapeutics for public use. However, the live-attenuated strain, TC-83, is used as a vaccine for military and at-risk personnel and the inactivated trivalent encephalitic virus is more commonly used in horses [30,31].

The general architecture of the alphavirus genome encodes for four nonstructural proteins (nsP1–4) and three major structural proteins (capsid, envelope proteins E1 and E2). In terms of basic functionality, the structural proteins interact to form the viral particle and the nonstructural proteins have critical roles in RNA synthesis and viral replication [32,33,34]. The only nonstructural protein that contains a large number of serine and threonine residues that could potentially be phosphorylated is nsP3 [29,34,35]. nsP3 among the alphaviruses varies in amino acid sequence and length. The protein is divided into an N-terminus that is highly conserved with viruses, bacteria and eukaryotes; an alpha domain that is conserved only among alphaviruses; and a hypervariable C-terminal domain (HVD) that shows no sequence homology among alphaviruses [29,34,36]. The function(s) of nsP3 has not yet been fully elucidated [29,32,34]; however, chimeric analysis, mutational and genetic studies of two prototypical old world alphaviruses: SINV and SFV have implicated a pathogenic role for nsP3 in infected mice [29] and a requirement for nsP3 in negative strand synthesis and subgenomic RNA replication [34].

Mass spectrometric analyses with on-target alkaline phosphatase digestion, as well as 2D peptide mapping and Edman sequencing were utilized to determine phosphorylated residues on nsP3 from SFV and SINV [35,37]. Electrophoretic mobility shifts indicated that SINV forms several species of phosphorylated nsP3 and may therefore be heavily phosphorylated [35,38]. In the case of SFV nsP3, multiple residues were determined to be phosphorylated: Ser320, Ser327, Ser332, and Ser335 and from 7 to 12 residues in peptide Glycine (Gly)338–Lysine (Lys)415 and Thr344 and/or Thr345 [39]. Within the HVDs of alphavirus nsP3s there are two acidic regions separated by a proline rich region. Most of the identified phosphorylated sites of SFV nsP3 were located in the first acidic region [35]. In an earlier report, Li *et al.*, implicated the host protein CK2 as phosphorylating SINV nsP3 [37]. CK2 recognizes Ser/Thr followed by acidic residues; however, pulse chase experiments analyzed by immunoprecipitation from lysates suggests that kinases other than CK2 could be responsible for phosphorylating SINV nsP3 [37]. In the case with SFV, Ser327 and Ser367 on nsP3 are CK2 phosphorylation sites; however alternate kinases may be available to phosphorylate these and other sites; such as, Ser320, Ser332, and Ser335 which are potential protein kinase C sites [35,38].

Although much of the studies on nsP3 have been focused on old world alphaviruses, research on new world alphavirus nsP3 is currently underway. For example, VEEV-nsP3 HVD contains 53 potentially phosphorylated residues [36]. Although phosphorylation of VEEV-nsP3 has not been validated by standard molecular biological techniques, mass spectrometry and confocal microscopy analyses identified and validated an association of VEEV-nsP3 with the host Inhibitor of nuclear factor kappa-B kinase subunit beta (IKKβ) [40]. Interestingly, this interaction was also visualized with WEEV-nsP3, suggesting a broad spectrum interaction of this host kinase with new world alphaviruses [40].

To ascribe functionality to phosphorylated nsP3 a number of *in vitro* and *in vivo* studies were performed. For example, a single mutation in the proline rich sequence motif in the HVD of SFV or SINV nsP3 greatly impaired RNA synthesis by disrupting binding with host amphiphysins [41]. In addition, deletions in SFV-nsP3 HVD decreased viral RNA synthesis in vertebrate cells such as, BHK-21 cells and BALB/3T3 cells [42]. In contrast, deletion of VEEV-nsP3 HVD did not affect viral replication in BHK-21 cells; however, in mosquito cells, the deletion rendered the virus replication incompetent. This suggests that the VEEV-nsP3 HVD functions in cell specificity [36]. Treatment of VEEV infected human astrocyte cells with the well documented IKKβ inhibitor, BAY-11-7082, resulted in inhibition of VEEV multiplication and disruption of the nsP3-IKKβ interaction [40], suggesting that the VEEV-nsP3 HVD plays a role in the viral life cycle. *In vivo* studies indicated that deletions in the HVD of SFV nsP3 resulted in reduced virulence in mice after peripheral and intra nasal inoculation [42]. The information gathered from these studies indicates an importance for the alphavirus nsP3 HVD in different aspects of the viral life cycle, such as viral RNA synthesis, virulence and/or protein-protein interactions. Therefore targeting the phosphorylation status of alphavirus nsP3 as a therapeutic option would not only be an effective strategy, but also be broad spectrum in nature, to restrict viral multiplication and increase host survival.

SINV is an old world alphavirus with a 49S genome which encodes four nonstructural proteins (nsP1–4) and three structural proteins (E1, E2 and capsid). The nonstructural proteins have been demonstrated to have critical roles in negative strand RNA synthesis and viral replication [43]. Among the nonstructural proteins, nsP3 is involved in the synthesis of negative-strands during the early phase of RNA replication. nsP3 is a phosphoprotein containing an N-terminal region highly conserved in alphaviruses and a non-conserved C terminal region. Posttranslational modification occurs on Serine and Threonine residues primarily in the C-terminal region involving kinases resembling CK2 and protein kinase C (PKC). Evidence has been shown that that degree of phosphorylation plays a role in the functioning of nsP3. Previous studies analyzing mutations of specific nsP3 amino acids in the macro-domain provide evidence that viral replication is disrupted as well as causing nsP3 instability in neurons. The mutation studies replacing amino acids at residue 68 of nsP3 was associated with decreased C-terminal nsP3 phosphorylation [38]. The reduced phosphorylation of nsP3 resulting from changes in the macrodomain shows the site to be important for recruiting of targeting kinases.

There are instances of SINV glycoprotein phosphorylation as being important for viral particle assembly. The report published by Lin and Brown utilized protein kinase inhibitors and infected baby hamster kidney (BHK) cells. Interestingly, the authors detected E2 phosphorylation in the presence of protein phosphatase inhibitors, suggesting that phosphorylated E2 is either of extremely transient occurrence or of low abundance. The authors suggest that differential phosphorylation and dephosphorylation of E2 was important for SINV particle maturation [44]. The authors, in a subsequent report, also demonstrated that the phosphorylation of E2 enabled interaction between E2 and the nucleocapsid protein [45].

CHIKV is garnering significant attention in recent times due to its introduction into the new world. Multiple laboratories are devoting time and effort to the development of novel vaccine and therapeutic candidates for the treatment of CHIKV infections. There are some interesting reports in the literature about host proteins being phosphorylated in CHIKV infections and the role of nsPs in combating the innate immune responses [46,47,48,49]. It would be interesting to determine if nsPs are phosphorylated in infected cells and if host protein kinase inhibitors can influence viral protein phosphorylation status.

SFV is an old world alphavirus with similar molecular and cellular biology to CHIKV. SFV has an 11.5 kb genome which encodes a non-structural and structural polyprotein [39]. The non-structural polyprotein is cleaved into four non-structural proteins (nsP1–4). Vihinen *et al.*, demonstrated that Thr344 and Thr345 on nsP3 are major phosphorylation sites. In knockdown experiments, these phosphorylation events were reported to have implications regarding viral RNA synthesis and pathogenicity [35]. It was recently demonstrated that nsP3, via its hyperphosphorylated C-terminal tail, potently activates the phosphatidylinositol-3-kinase (PI3K)-protein kinase B (PKB, also called Akt)-mammalian target of Rapamycin (mTOR) pathway, a pro-survival signaling cascade. SFV and CHIKV both activate the PI3K-Akt-mTOR pathway, but SFV is a more potent and persistent activator of Akt than CHIKV. It was shown that the hyperphosphorylated tail of SFV is responsible for this induction, and the region could be functionally transferred to CHIKV. Akt activation was linked to efficient internalization of replication complexes in SFV, but not CHIKV, and could have implications in pathology [50].

#### 3.1.3. Rubivirus

Rubella virus is the only member of the genus Rubivirus, family *Togaviridae*, with virions composed of three structural proteins; two membrane-spanning glycoproteins E2 and E1, and a capsid protein. Replication complexes are formed following viral entry, and are closely associated with the ER, mitochondria, and Golgi. The capsid protein interacts with viral genomic RNA and both glycoproteins during viral assembly, in order to support nucleocapsid formation and viral budding. Formation of rubella nucleocapsid is membrane dependent through post-translational processing of the capsid protein. Data shows that phosphorylation of Ser46 within the RNA binding site of the capsid regulates the interaction between the capsid and genomic RNA, as well as subsequent phosphorylation of other amino acid residues within the RNA binding site [51,52]. Capsid protein has also been shown to interact with the mitochondrial matrix protein p32, causing mitochondrial redistribution in infected cells, blocking mitochondrial transport, and inhibiting apoptosis [52]. Additional studies have demonstrated that Ser48, Ser52, and Thr47 of the RNA binding site are targeted for phosphorylation [53]. Capsid phosphorylation in Rubella virus was critical for viral replication, but not required for virion assembly. Hypophosphorylation of capsid results in nucleocapsid disassembly leading to replication defects. The kinase responsible for capsid phosphorylation is not yet known; however, it would be interesting to determine the impact of capsid protein phosphorylation on mitochondrial localization and mitochondrial dynamics as a reflection of host survival fate.

### 3.2. Negative Sense ssRNA Viruses

#### 3.2.1. Filovirus

Ebola virus (EBOV) is a member of the *Filoviridae* family, The EBOV particle is composed of seven structural proteins, of which five proteins contribute to the nucleocapsid. The ribonucleoprotein complex, which mediates transcription and replication of EBOV genome, is composed of NP, VP35, VP30, VP24, and L protein. Viral polymerase L is an enzymatically active protein, which is bound to the nucleocapsid via polymerase cofactor VP35. VP24 and VP40 are matrix proteins associated with the regulation of viral genome replication, transcription, and viral egress.

VP30, a zinc-finger protein, initiates transcription of the first gene as well as subsequent genes that follow, however it is not required for viral replication [54,55]. VP30 activity is regulated by its phosphorylation, which is modulated at the two N-terminal serine clusters consisting of three serine residues per cluster (Ser29, 30, 31 and Ser42, 44, 46). Mutation of serine residues to alanine (mimicking nonphosphorylated serine) or to aspartate (mimicking weakly phosphorylated serine) supports initiation of viral transcription, but fully phosphorylated VP30 does not. Whether VP30 phosphorylation results in direct effect on replication or an indirect effect of down regulation of viral transcription has yet to be discovered. It is apparent, however, that VP30 phosphorylation acts as a molecular switch for diverse functions of the protein; non-or weakly phosphorylated VP30 promotes transcription until it is removed by phosphorylation-induced binding to nucleocapsid. VP30 dephosphorylation, mediated by host protein phosphatase 1 (PP1), is important for mediating transcriptional function of EBOV RdRp [56,57].

VP40 is known to play a role in the morphogenesis of filamentous virions and their successive budding. Studies using *in vitro* models that recapitulate aspects of Ebola infections demonstrated that Abelson murine leukemia viral oncogene homolog 1 (ABL1), a tyrosine kinase, regulates formation of VLP’s and stimulates replication of EBOV in cells. This conclusion was made by using specific Small interfering RNA (siRNA)’s, pharmacological inhibitors (nioltinib and imantinib), and Abelson murine leukemia viral oncogene homolog 1 (cABL1)+/− knockout cell lines. c-ABL1 was found to contribute to the phosphorylation of five tyrosine residues of VP40, which regulated EBOV budding from infected cells. Nucleocapsid formation is not dependent on c-ABL1 but the kinase does regulate the transport of nucleocapsid to the membrane and virion release, which are both dependent on VP40. The regulation of this process through tyrosine phosphorylation represents interaction between host and viral proteins which may be used as therapeutic intervention strategies [58].

#### 3.2.2. Orthomyxovirus

Influenza viruses belong to the family *Orthomyxoviridae* and cause seasonal epidemics and occasional pandemics. Influenza A virus has a segmented genome consisting of eight segments encoding 11 viral proteins. The influenza A virus nonstructural protein NS1 is a multifunctional virulence factor which aids viral replication by modulating viral RNA metabolism, host innate immune responses, and cell signaling pathways. NS1 is also believed to affect double stranded RNA (dsRNA) binding capacity through phosphorylation of Thr215, Ser42, and Ser48 [59,60]. Thr215 is a known phosphorylation site of the NS1 protein with members of both cyclin dependent kinases (CDK) and extracellular signal-regulated kinases (ERK) families responsible for modification *in vitro*, however the biological function of this phosphorylation is unknown. Recent studies have shown a functional interaction between NS1 with Akt, an intercellular survival regulator kinase. The RNA-binding domain was shown to interact with Akt, increasing kinase activity, which resulted in the phosphorylation of NS1 at the Thr215 residue. Akt inhibitors are therefore, considered to be appropriate targets for therapeutics against viral infection. The Akt inhibitor TCL-1, was effective in inhibiting kinase activity, suppressing viral entry and viral genome replication [61,62]. Additionally, mutation of Ser42 eliminating interaction of NS1 with dsRNA and attenuating viral multiplication [60,61].

Another phosphorylation site of Influenza A virus identified through mass spectrometry is Tyr132 of the matrix protein (M1). After entry of the virus, the M1 protein undergoes a pH-dependent conformational change, which releases the viral ribonucleoproteins into the cytoplasm. The M1 protein can inhibit viral transcription, which suspends viral RNA synthesis and begins to promote replication during the late phase of infection. This phosphorylation of Tyr132 is essential to viral replication by controlling the nuclear import of M1. M1 also contains both phosphoserine and phosphothreonine residues with the potential to be phosphorylated by PKC and extracellular signal-regulated protein kinases [62,63]. AG490, a Janus kinase 2 (JAK2) inhibitor, prevented the nuclear import of M1 during viral replication which was further supported through genome analysis showing JAK2 having potential interaction with M1. Further investigation is required to confirm the role of the kinase involved, the effects of proteins which recognize phosphorylated M1, and their specific roles in the downstream pathways. Blocking of this interaction can disrupt the bond between virus and host cell providing a possible therapeutic strategy [64,65].

Influenza nucleoprotein (NP) is known to be a serine rich protein, particularly on the *N*-terminus [66]. Inhibition of NP phosphorylation results in its nuclear retention. Mutation of conserved phosphorylation sites including Ser9, Tyr10 and Ser165 results in decreased viral titers [65]. Phosphorylation of Ser9, Tyr10 and Tyr296 was also important for *in vivo* viral multiplication [67]. Interaction of NP with host importin-α was regulated by Ser9 and Tyr10 phosphorylation while Tyr296 phosphorylation moderated interaction between NP and chromosomal maintenance 1 (CRM1) [68].

## 4. Viral Protein Phosphorylation Using Host Enzymes—Strength or Weakness?

Viruses have to evolve continuously and evolution is largely directed by the pressures imposed on them by the host which has an impact on both the host and the pathogen. The co-evolution process exerts a reciprocal selection pressure on both the pathogen and the host; however, the consequences become more apparent in the case of the pathogen, in this case, viruses, because of their shortened multiplication time and an exaggerated population/progeny size. This makes it possible for us to understand evolutionary impact on specific portions of viral genomes when a selection pressure is applied in the form of an inhibitory small molecule compound. It is beginning to be more and more appreciated that there are overlaps in the host responses to diverse infectious agents. So, an important challenge in the field of emerging infectious diseases is to define the critical targets and network interactions that are subverted/altered/modified/leveraged by acutely infectious viruses to elicit a progressive infection in the host.

Viral proteins, particularly those that are transiently post translationally modified, provide a discerning and powerful set of tools to understand both how the host interfaces with the pathogen. They also offer insights into how a limited set of pathogen components such as a handful of viral genes can manipulate a complex, multi-tiered, redundantly wired system as the human cell. Post translational modification can contribute to increasing affinity of protein:protein interactions, stabilize interactions and skew the availability of kinases in favor of the pathogen. It seems to be a powerful strategy adopted by viruses to establish an efficacious multiplication cycle. In enabling such intricately regulated processes to counter the host response, the viruses also expose unique weaknesses that can be exploited, particularly by developing targeted therapeutics. Often, it may not be necessary to develop a therapeutic that inhibits the target kinase of a viral protein, but we may be able to target upstream effector molecules as many of the activated kinases are results of signal transduction events.

A prominent gap in our understanding of viral life cycle for many of these pathogens is little to no information on the specific post translational modifications on viral proteins that drive protein:protein interactions. With the current developments in the field of proteomics, it is not inconceivable to specifically address phosphorylation of viral proteins in infected cells and the impact of those modifications on protein interaction networks. It is not obvious that phosphorylation of residues is linearly related to multiplicity of infection. In fact, it is more than likely that different phosphorylation events may be recorded as variables of both time post infection and multiplicity of viral infection. It is also possible to record viral protein phosphorylation in the context of efficacious inhibitors using similar strategies. It would not be surprising if inhibitor treatment results not necessarily in abrogation of all of the phosphorylation targets, but only a select few. Such modified residues will further enable us to fine tune interaction domains of the viral proteins with host proteins. Delineation of susceptible phosphorylation targets will also pave the way for rational design of live attenuated viruses for use as vaccines by targeted mutations in the viral genomes.

## 5. Repurposing of FDA Approved Therapeutics

The search for therapeutics for the treatment of all of the above mentioned infectious diseases is aggressively ongoing with some notable efforts made in the realm of novel small molecule therapeutics. An accepted caveat to this uphill task is that most of the novel candidate inhibitors that show efficacy in the early preclinical discovery stages do not complete the entire drug development pipeline. It is not uncommon to find therapeutic candidates dropping off the development pipeline during phased trials. An additional pitfall that we often encounter is the development of pathogen resistance to small molecule inhibitors that target the pathogen itself. The example of influenza and resistance to Amantadine is all too well known [69,70,71]. Therefore, to counteract the dual concern of candidate therapeutic failure during drug development and emergence of pathogen resistance, the repurposing of drugs that have been currently FDA approved for alternate indications is being pursued by many laboratories. The ability to target host-based events enables us to not only circumvent the issue of pathogen resistance, but also provides the opportunities to develop broad spectrum therapeutics that may be utilized against multiple infectious agents. Currently, the primary host-based broad spectrum therapeutic candidate that is commonly used is Ribavirin.

The recent Ebola outbreak has shed light on the reality of an urgent need for effective therapeutics. Johanesen *et al.*, have reported the results from a screen of 2600 FDA-approved inhibitors for efficacy against Zaire Ebola virus. They have identified multiple classes of FDA-approved inhibitors including selective estrogen receptor modulators, antihistamines, calcium channel blockers, and antidepressants. The authors also demonstrated *in vivo* relevance of their results in a mouse model and suggest that most of the effective inhibitors are blockers of late stages of viral entry [72]. The same research group had reported a couple of years earlier that selective estrogen receptor modulators (SERMs), including clomiphene and toremifene, were potent inhibitors of EBOV infection as demonstrated in a mouse model [72]. An independent screen of 1014 FDA-approved drugs for off-label broad-spectrum efficacy against multiple biothreat agents including Ebola virus, Marburg virus and Lassa virus was conducted by Madrid *et al.*, and the authors have demonstrated the usefulness of Chloroquine as an antiviral, particularly using *in vitro* and *in vivo* models of Ebola virus infection [73]. Studies performed by Kouznetsova *et al.*, using a novel 1536-well plate assay to screen for entry inhibitors of Ebola virus-like particles (VLPs) containing the glycoprotein (GP) and the matrix VP40 protein fused to a beta-lactamase reporter protein which could be utilized in biosafety level 2 laboratories to screen libraries for antiviral compounds. The authors demonstrated that 53 FDA-approved inhibitors that included microtubule inhibitors, estrogen receptor modulators, antihistamines, antipsychotics, pump/channel antagonists, and anticancer therapeutics were effective against Ebola virus [74].

Repurposing of FDA-approved therapeutics has also been extended to alphaviruses. In a recent publication, the efficacy of bortezomib, the host-based proteasome inhibitor, was illustrated in the context of new world alphaviruses. Inhibition of the host proteasome was shown to influence the ubiquitination status of capsid as a potential mechanism of action [75]. It is however possible and fully anticipated that additional mechanisms will contribute to the inhibition mediated by bortezomib that transcends ubiquitination. For example, proteasome inhibition will directly impact the NFkB cascade which will quickly metastasize in its impact on cellular signal transduction and influence multiple signaling events. Voss *et al.*, have also demonstrated the relevance of the MEK/ERK signaling pathway to new world alphavirus multiplication. The MEK1/2 inhibitor GSK1120212, which has already been FDA-approved, was shown to be an efficacious inhibitor of VEEV[76]. Amaya *et al.*, utilized the kinase inhibitor BAY-11-7082, an anti-inflammatory agent which inhibits phosphorylation of nuclear factor of kappa light polypeptide gene enhancer in B-cells inhibitor alpha (IκBα), to potently inhibit VEEV [40]. The rationale behind that approach was that BAY-11-7082 also impacted phosphorylation of viral target proteins, with nsP3 being a potential candidate. Ongoing studies in our laboratory continue to examine the impact of MEK/ERK inhibitors and inhibitors of IKKβ functionality for influence on VEEV protein post translational modification.

It would be an interesting approach to test some of the effective candidates in a sub-effective dose study to identify specific residues on the viral proteins that are targeted by the inhibitors and if any of those residues are subjected to post translational modification by host kinases. Such a strategy was effectively utilized by Chung *et al.*, where the authors had discovered a novel compound with antiviral activity against VEEV and identified that the compound influenced nsP2 function [77]. nsP2 is the VEEV protease and is likely to be impacted by its phosphorylation status. Such an approach could very well be extended to the FDA approved inhibitors that show efficacy in appropriate experimental systems. Novel approaches like high content imaging strategies can shed light on intracellular localization effects of many of these effective candidate inhibitors on viral proteins [78].

Flaviviruses, particularly DENV, infects such a large portion of the human population on an annual basis with a prominent number of hospitalizations that the search for a vaccine and therapeutic has been aggressive. Vaccine development for DENV has been particularly challenging because of the requirement to be equivalently efficacious against all four DENV serotypes. In the search for small molecule inhibitors for the treatment of DENV infection, Cheung *et al.*, adopted a similar strategy of screening a FDA-approved drug library and report that lanatoside C, an FDA approved cardiac glycoside as an efficacious anti-DENV compound. The authors suggest that lanatoside C was likely to inhibit the early processes of DENV infectious cycle. They also demonstrated that the compound had a broad spectrum antiviral activity against multiple positive sense ssRNA viruses including Kunjin Virus (flavivirus), Chikungunya virus (alphavirus), SINV (alphavirus) and human enterovirus 71 [79]. Additional studies have indicated the utility of kinase inhibitors that would interfere with flaviviral NS5 function. Small molecule mediated inhibition of casein kinase was shown to suppress YFV multiplication, with efficacy against HCV NS5A protein [80]. While the Quintavalle *et al.*, study utilizes non-FDA approved casein kinase inhibitors, FDA approved alternatives, such as pyrvinium, have been shown to potently inhibit casein kinase 1 alpha (CK1α) and could be investigated in the context of flaviviral infection [81]. This is a great example of how host-based small molecule inhibitors can have utility against multiple viruses which utilize common host pathways.

As detailed in multiple instances earlier, phosphorylation of viral proteins plays an important role in intracellular transport and distribution. It may very well be that at least a fraction of these inhibitors may impact viral protein localization. A similar high content strategy was utilized to screen for inhibitors of DENV using a FDA approved drug library which identified multiple transporters, receptors and protein kinases as being targets of effective inhibitors [82]. Mass spectrometry based phosphoproteomics approaches, using protein lysates from infected, treated *versus* infected, untreated cells will be exceptionally informative in identifying the impact of these host-based inhibitors on both the host proteome and the viral proteome. The reason why the analysis of the host proteome may carry significance is that, when a cell is infected, we anticipate a change in the proteomic landscape which will unravel novel targets for therapeutics that do not necessarily conform with the anticipated mechanism of action associated with a compound. This was shown to be the case in many instances, one of which was the demonstration of antiviral efficacy of an antioxidant small molecule compound NSC62914 by Panchal *et al.*, with broad spectrum efficacy against filoviruses, alphaviruses and arenaviruses. When the authors tested other antioxidant molecules, the efficacy did not parallel that of NSC62914 leading the authors to propose that alternate mechanisms, possibly involving host kinases were likely to be involved [83].

## 6. Conclusions

This review has attempted to draw attention to the post translational modification of virally derived proteins, with an emphasis on phosphorylation. Viruses, being the perfect parasites, have exquisitely learnt to adapt to the innate immune responses that are mounted by the host cell upon infection. Their high multiplicity potentials and short time of infectious cycles permit them to carry out their evolutionary experiments on an extremely compressed time scale that for every push from the cell, there is a well-balanced pull from the virus. It makes it almost obvious that viruses are going to utilize pre-activated kinases (as part of innate immunity) to their advantage and identify ways to engage the kinase to augment their own multiplication by enhancing viral transcription, viral protein processing and/or particle assembly. Post translational modification of viral proteins also contributes by intricately and exquisitely regulated hijack, camouflage, seek-and-destroy mechanisms that protect the virus from the innate immune host responses, which have not been discussed in this review. The objective has been to emphasize the importance of these post translational modification events to viral multiplication, which makes them not only their strength, but also their weakness that can be exploited for the development of efficacious therapeutics. In silico prediction approaches have evolved to the extent where modification sites, effector enzymes, consequences of protein modification (interactions with protein partners) can be predicted by various kinds of machine learning tools to a large extent that can pinpoint viral targets, reduce the need for large screens and contribute to the success of the FDA-approved drug repurposing efforts. Viral protein phosphorylation, hence, can offer novel mechanistic insights into the intricate interactions between the host and the pathogen during an acute viral infection and also provide a strong rationale for the development of effective therapeutics.
